# A semi-simulated EEG/EOG dataset for the comparison of EOG artifact rejection techniques

**DOI:** 10.1016/j.dib.2016.06.032

**Published:** 2016-06-29

**Authors:** Manousos A. Klados, Panagiotis D. Bamidis

**Affiliations:** a Max Planck Institute for Human Cognitive and Brain Sciences, Leipzig 04103, Germany; b Lab of Medical Physics, School of Medicine, Aristotle University of Thessaloniki, Thessaloniki, Greece

**Keywords:** EEG, EOG, Artifact Rejection

## Abstract

Artifact rejection techniques are used to recover the brain signals underlying artifactual electroencephalographic (EEG) segments. Although over the last few years many different artifact rejection techniques have been proposed (http://dx.doi.org/10.1109/JSEN.2011.2115236[1], http://dx.doi.org/10.1016/j.clinph.2006.09.003[2], http://dx.doi.org/10.3390/e16126553[3]), none has been established as a gold standard so far, because assessing their performance is difficult and subjective (http://dx.doi.org/10.1109/ITAB.2009.5394295[4], http://dx.doi.org/10.1016/j.bspc.2011.02.001[5], http://dx.doi.org/10.1007/978-3-540-89208-3_300. [6]). This limitation is mainly based on the fact that the underlying artifact-free brain signal is unknown, so there is no objective way to measure how close the retrieved signal is to the real one. This article solves the aforementioned problem by presenting a semi-simulated EEG dataset, where artifact-free EEG signals are manually contaminated with ocular artifacts, using a realistic head model. The significant part of this dataset is that it contains the pre-contamination EEG signals, so the brain signals underlying the EOG artifacts are known and thus the performance of every artifact rejection technique can be objectively assessed.

**Specifications Table**

**Table:** 

Subject area	*Neuroscience*
More specific subject area	*EEG Artifact Rejection*
Type of data	*EEG and EOG signals in MATLAB arrays, links*
How data was acquired	*Electroencephalography, Electrooculography*
Data format	*Filtered*
Experimental factors	*Filtering, and artificially contaminated with EOG signals*
Experimental features	*This dataset can be used to assess the performance of an artifact rejection technique.*
Data source location	*Thessaloniki, Greece*
Data accessibility	*Data is within this article and is downloadable without restriction using the following URL:**https://data.mendeley.com/datasets/wb6yvr725d/1*

**Value of the data**

• First EEG dataset artificially contaminated with EOG artifacts using a realistic contamination model.
• Subjective assessment of EOG artifact rejection technique.
• Serves as a reference point for the scientific community in order to compare various algorithms in the same dataset.

## 1 Data

This work presents a semi-simulated EEG dataset, where artifact-free EEG signals are manually contaminated with ocular artifacts following the model proposed by [7]. The significant part of this dataset is that it contains the pre-contamination EEG signals, so the brain signals underlying the EOG artifacts are known and thus the performance of every artifact rejection technique [1], [2], [3] can be objectively assessed [4], [5], [6]. The main differences of the proposed dataset compared to others (p.e. see [8], [9]) is that it is focused only on EOG artifacts, using a realistic model for the contamination of artifact-free EEGs and not a random procedure.

The data are available for downloading without any restriction using this URL:

https://data.mendeley.com/datasets/wb6yvr725d/1.

## 2 Experimental design, materials and methods

EEG data were obtained from twenty-seven healthy subjects, 14 males (mean age: 28.2 ± 7.5) and 13 females (mean age: 27.1±5.2), during an eyes-closed session. Nineteen EEG electrodes (FP1, FP2, F3, F4, C3, C4, P3, P4, O1, O2, F7, F8, T3, T4, T5, T6, Fz, Cz, Pz) were placed according to the 10–20 International System, with odd indices referenced to the left and even indices to the right mastoid respectively, while the central electrodes (Fz, Cz, Pz) were referenced to the half of the sum of the left and right mastoids. Signals’ sampling frequency was 200 Hz and a band pass filtered at 0.5–40 Hz and notch filtered at 50 Hz were applied. From these twenty-seven subjects we have obtained fifty-four datasets in total and each one has 30 s duration. The obtained datasets were carefully inspected in order to ensure that there is no significant contamination by biological or external artifacts.

Moreover, EOG signals were obtained from the same subjects, during an eyes-opened condition, using four electrodes placed above and below of the left eye and another two on the outer canthi of each eye. This process gave rise to two bipolar signals, namely, vertical-EOG (VEOG), which is equal to the upper minus lower EOG electrode recordings and horizontal-EOG (HEOG), which is equal to the left minus right EOG electrode recordings. These EOG signals were band-pass filtered at 0.5–5 Hz [10].

In order to produce this semi-simulated EEG dataset, we used the contamination model proposed by [7] and it follows the next equation:

$${\mathrm{Contaminated}\_\mathrm{EEG}}_{i,j}={\mathrm{Pure}\_\mathrm{EEG}}_{i,j}+a_{j}\mathrm{VEOG}+b_{j}\mathrm{HEOG}$$

where Contaminated_EEG are the artificially contaminated EEG signals and Pure_EEG are the signals obtained during the eyes-closed session. The VEOG and HEOG are the EOG signals that were previously described, while vectors $a_{j}$, $b_{j}$ describe the contamination coefficients for VEOG and HEOG, respectively, initialized according to [10]. Finally the index i indicates the subject’s number, while jdenotes the electrode’s number.

The contamination coefficients $a_{j}$, $b_{j}$ were computed for each subject separately. For each subject, EEGs and EOGs were taken from an eyes-opened session. The VEOG signals were used to detect all the blink segments (start – peak – end) and, after confirming that in the same segments the EEGs are also corrupted by blinks, we used linear regression among their amplitudes to compute the $a_{j}$. The same procedure was also applied in HEOGs for the $b_{j}$ computation, with the only difference that the input to linear regression was the amplitude of the horizontal plateaus generated by the horizontal eye movements.
